# Toxicity of the 3,4-Methylenedioxymethamphetamine and Its Enantiomers to *Daphnia magna* after Isolation by Semipreparative Chromatography

**DOI:** 10.3390/molecules28031457

**Published:** 2023-02-02

**Authors:** Ana Rita Costa, Virgínia M. F. Gonçalves, Bruno B. Castro, João Soares Carrola, Ivan Langa, Ariana Pereira, Ana Rita Carvalho, Maria Elizabeth Tiritan, Cláudia Ribeiro

**Affiliations:** 1 TOXRUN-Toxicology Research Unit, University Institute of Health Sciences, IUCS-CESPU, CRL, 4585-116 Gandra, Portugal; 2 UNIPRO-Oral Pathology and Rehabilitation Research Unit, University Institute of Health Sciences (IUCS), CESPU, 4585-116 Gandra, Portugal; 3 CBMA (Centre of Molecular and Environmental Biology), Department of Biology, University of Minho, 4710-057 Braga, Portugal; 4 Institute of Science and Innovation for Bio-Sustainability (IB-S), University of Minho, 4710-057 Braga, Portugal; 5 Department of Biology and Environment, University of Trás-os-Montes and Alto Douro, Quinta de Prados, 5000-801 Vila Real, Portugal; 6 Centre for the Research and Technology of Agro-Environmental and Biological Sciences (CITAB), University of Trás-os-Montes and Alto Douro (UTAD), Quinta de Prados, 5000-801 Vila Real, Portugal; 7 Interdisciplinary Center of Marine and Environmental Research (CIIMAR), University of Porto, 4450-208 Matosinhos, Portugal; 8 Laboratory of Organic and Pharmaceutical Chemistry, Department of Chemical Sciences, Faculty of Pharmacy, University of Porto, 4050-313 Porto, Portugal

**Keywords:** aquatic organisms, chirality, ecotoxicity, enantioselectivity, psychoactive drugs

## Abstract

MDMA (3,4-methylenedioxymethamphetamine) is a chiral psychoactive recreational drug sold in illicit markets as racemate. Studies on the impact of MDMA on aquatic organisms are scarce. While enantioselectivity in toxicity in animals and humans has been reported, none is reported on aquatic organisms. This study aimed to investigate the ecotoxicological effects of MDMA and its enantiomers in *Daphnia magna*. For that, enantiomers (enantiomeric purity > 97%) were separated by liquid chromatography using a homemade semipreparative chiral column. Daphnids were exposed to three concentrations of (*R*,*S*)-MDMA (0.1, 1.0 and 10.0 µg L^−1^) and two concentrations of (*R*)- and (*S*)-enantiomers (0.1 and 1.0 µg L^−1^) over the course of 8 days. Morphophysiological responses were dependent on the substance form and daphnia development stage, and they were overall not affected by the (*R*)-enantiomer. Changes in swimming behaviour were observed for both the racemate and its enantiomers, but enantioselective effects were not observed. Reproductive or biochemical changes were not observed for enantiomers whereas a significant decrease in acetylcholinesterase and catalase activity was noted at the highest concentration of (*R*,*S*)-MDMA (10 µg L^−1^). Overall, this study showed that sub-chronic exposure to MDMA racemate and its enantiomers can interfere with morphophysiological and swimming behaviour of *D. magna*. In general, the (*R*)-enantiomer demonstrated less toxicity than the (*S*)-enantiomer.

## 1. Introduction

Psychoactive substances (PASs) have been widely reported as environmental contaminants [1,2]. MDMA (3,4-methylenedioxymethamphetamine) is a chiral PAS synthetic derivative of amphetamine ranking among the most consumed recreational drugs in Europe [3,4]. MDMA has a stimulating action on the central nervous system and exhibits enantioselective effects; the (*S*)-enantiomer has been shown to be more potent than the (*R*)-enantiomer in producing euphoria, energy and a desire to socialize [5,6]. In 2017, the Multidisciplinary Association for Psychedelic Studies supported a clinical trial for possible Food and Drug Administration approval of the use of MDMA for the treatment of post-traumatic stress disorder (PTSD). The trial has recently progressed to the second stage of Phase 3 and all trials so far have shown promising results in mitigating PTSD [7,8]. The study is estimated to end soon and the therapy could be implemented in 2023 or 2024 [8,9].

MDMA is sold in illicit markets in the form of a racemate (50% of *R* and *S* enantiomers) but its metabolism is enantioselective [6]. (*S*)-MDMA is more rapidly metabolized, leading to an enrichment of the (*R*)-enantiomer in urine and later in the aquatic environment [2,10]. The processes of biological degradation in wastewater treatment plants are also enantioselective, with consequent discharge into receiving systems at different enantiomeric fractions [2,10]. Therefore, enantiomers should not be neglected when addressing hazards and environmental risk assessment of MDMA. For instance, developmental neurotoxic effects of MDMA were reported in zebrafish embryos but enantioselective effects were not studied [11]. However, previous studies from our group [12,13] have shown enantioselective responses of aquatic organisms to various PASs, including amphetamine (Ribeiro et al., submitted).

Chemical analysis of wastewater from 42 European cities in 2017 and 2018 revealed an increase in the prevalence of MDMA detection, which points to an increase in the consumption of this substance and/or its purity [4]. Additionally, MDMA removal rates in wastewater treatment plants (WWTPs) are generally poor, ranging from 12% to 88%, with frequent detection in surface and drinking water [14,15]. Considering the possible approval of MDMA-assisted therapy and its low degradation rates, an increase in its occurrence in the environment is expected. As such, the eco-pharmacovigilance and the assessment of potential environmental risks of MDMA and its enantiomers are of utmost importance. However, studies on the impact of MDMA on aquatic organisms are scarce and its enantioselective toxicity on non-target organisms was never reported before.

To address the above knowledge gaps, this study aimed to assess the ecotoxicological effects of MDMA and its enantiomers in a model freshwater crustacean (*Daphnia magna*), targeting potential enantioselective responses. To do so, we conducted the enantioseparation of MDMA using semipreparative chromatography and investigated the biological responses of *D. magna* to racemate and single enantiomers in a critical ontogenetic period [16,17] of this model organism. *Daphnia magna* is an ecologically relevant organism widely used in ecotoxicological studies due to its short life cycle, fecundity, easy manipulation and high sensitivity to a variety of chemicals [18,19].

## 2. Results and Discussion

MDMA is sold in illicit markets in the racemate form and pure enantiomers are not available. Thus, in this study, the methodology optimized by Gonçalves et al. (2019) was applied to isolate the enantiomers for further use in ecotoxicity assays [20].

Many parameters were considered as checkpoints of toxicity to give a thorough understanding of the potential impact and enantioselective effects of MDMA. The chosen MDMA concentrations were based on reported levels for surface waters and wastewaters (0.1 μg L^−1^ to 1 μg L^−1^) and a worst-case exposure scenario, at a sub-lethal concentration, was included (10 μg L^−1^) for the racemic form. Because there may be differences in sensitivity across daphnia ontogenetic phases, the experimental design was carried out by choosing specific days for the determination of each of the parameters including the important daphnia developmental stages (transition from juvenile to adult) [21].

### 2.1. Multimilligram Enantioresolution of MDMA

#### 2.1.1. Injection Volume Optimization and Enantioseparation

**Figure 1:**
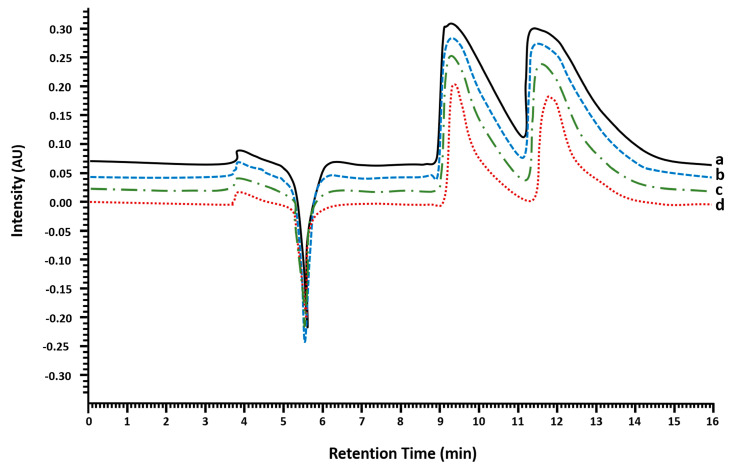
Chromatogram showing the injection volume optimization for the enantioseparation of (*R*,*S*)-MDMA in a semipreparative amylose 3,5-dimethilphenylcarbamate column by LC-DAD at normal elution mode. Mobile phase: *n*-Hex (0.1% DEA) and EtOH (0.1% DEA), 80:20 *v*/*v*; flow rate: 1.5 mL min^−1^; detector: 210 nm. Standard solution at 30 mg mL^−1^ (EtOH). Injection volume: Line (a) 20 µL; line (b) 15 µL; line (c) 10 µL; and line (d) 5 µL.

The semipreparative enantioseparation of MDMA was performed by liquid chromatography with diode array detection (LC-DAD) using a homemade semipreparative chiral column. The injection volume was optimized for the enantiomeric separation of (*R*,*S*)-MDMA using a stock of 30 mg mL^−1^ in ethanol (EtOH). The strategy consisted of studying different injection volumes considering the column overload capacity to make the process as profitable as possible in terms of purity, yield and number of injections while maintaining a good resolution. Figure 1 shows the chromatograms with 5, 10, 15 and 20 µL of injection. The optimized conditions for enantioseparation were established with an injection volume of 20 µL at 30 mg mL^−1^.

**Figure 2:**
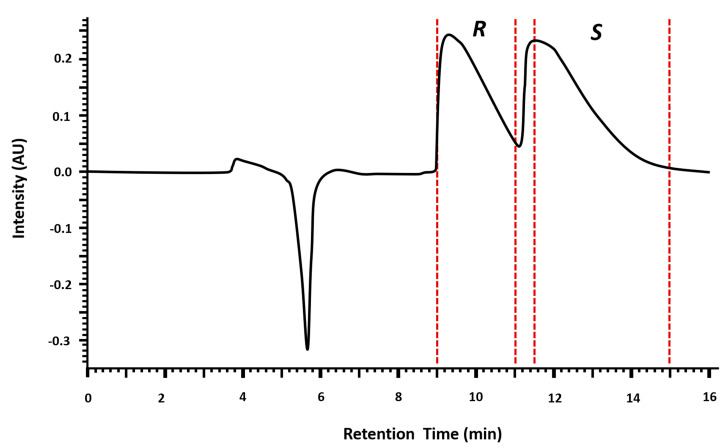
Chromatogram showing the enantioseparation of MDMA using a semipreparative amylose 3,5-dimethilphenylcarbamate column by LC-DAD. Mobile phase: *n*-Hex (0.1% DEA) and EtOH (0.1% DEA), 80:20 *v*/*v*; flow rate: 1.5 mL min^−1^; detector: 210 nm; injection volume: 20 µL of solution at 30 mg mL^−1^ (EtOH). The red dotted line corresponds to the cut-off time, that is, the time when each enantiomeric fraction was collected. (*R*)-MDMA was collected from 9 min to 11 min, the intermediate fraction was collected from 11 min to 11.5 min and (*S*)-MDMA was collected from 11.5 to 15 min.

Figure 2 shows the time of fractions collection: (*R*)-MDMA enantiomer was first eluted and collected from 9 to 11 min and (*S*)-MDMA was collected from 11.5 to 15 min. An intermediate fraction was collected (from 11 to 11.5 min) to assure high enantiomeric purity. The intermediate fraction was concentrated and re-injected in a volume of 100 µL.

#### 2.1.2. Enantiomeric Purity and Recovery of the Enantiomers

Enantiomeric purity of (*R*)-MDMA was higher than 99% and (*S*)-MDMA was achieved with 97% of enantiomeric purity.

**Figure 3:**
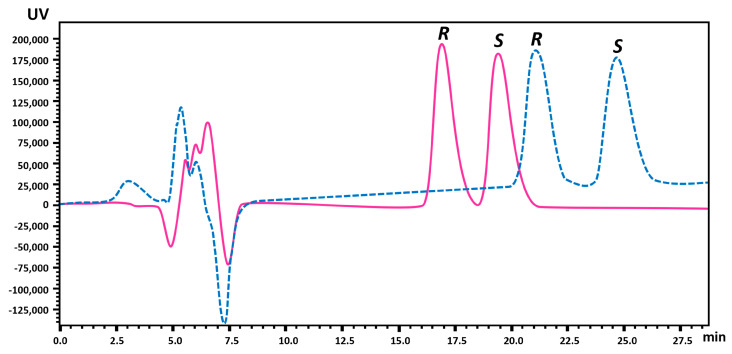
Chromatogram showing the separation of MDMA enantiomers ((*R*)-MDMA, first enantiomer, and (*S*)-MDMA, second enantiomer) in the analytical column (Lux^®^ 3µm i-Amylose-3 column) in reversed elution mode. Mobile phase: EtOH and UPW with 0.1% DEA; flow rate: 0.1 mL min^−1^; detector: 210 nm; injection volume: 10 µL. Standard solution at 100 µg mL^−1^ (EtOH). Legend: Dashed line—EtOH and UPW with 0.1% DEA (65:35, *v*/*v*), and solid line—EtOH and UPW with 0.1% DEA (70:30, *v*/*v*).

The amount of enantiomers obtained from the semipreparative method was quantified using the Lux^®^ (Kolkata, India) 3 µm i-Amylose-3 column (LC Column 150 × 2.0 mm) with EtOH and ultrapure water (UPW) with 0.1% DEA as mobile phase. In order to achieve the best chromatographic performance (low retention time while maintaining good resolution) for analytical purposes, the mobile phase was tested at different proportions of EtOH and UPW (65:35 and 70:30 *v*/*v*) (Figure 3). The optimized conditions were established with EtOH and UPW with 0.1% DEA (70:30, *v*/*v*) as mobile phase, a flow rate of 0.1 mL min^−1^, a wavelength of 210 nm and an injection volume of 10 µL (Rs = 1.5).

The analytical method was validated to quantify the enantiomers. The calibration curves were found to be linear in the range of 5.0 to 50 μg mL^−1^ with an R^2^ greater than 0.9996 for the (*R*)-enantiomer and in the range of 5.0 to 75 μg mL^−1^ with an R^2^ equal to 0.9999 for the (*S*)-enantiomer (calibration curve and linear parameters can be found in Table S2 of Supplementary Materials).

As the initial amount of MDMA was 30 mg mL^−1^, it would be expected to obtain 15 mg of each enantiomer in the semi-preparative isolation. However, the recovery percentage was 40.7% for (*R*)-MDMA and 2.0% for (*S*)-MDMA. The low percentage of recovery for the (*S*)-enantiomer could be related to the loss of substance during the crystallization process to obtain the hydrochloride salt. Nevertheless, the methodology allowed to obtain the isolated enantiomers with high enantiomeric purity (>97%), which allowed to proceed with the enantioselective ecotoxicity assays.

### 2.2. Ecotoxicity Assays

In order to certify the concentrations of culture media, a previously described analytical enantioselective gas chromatography mass spectrometry (GC-MS) method was adapted to quantify MDMA enantiomers [2]. The method was linear over the selected range of concentrations (R^2^ > 0.99) and used for quantification. For the nominal concentrations of (*R*,*S*)-MDMA of 0.1, 1 and 10 μg L^−1^, measured concentrations were 0.093, 0.90 and 9.35 μg L^−1^, respectively. For the enantiomers, nominal concentrations of 0.1 and 1 μg L^−1^ corresponded to a measured concentration of 0.090 and 0.98 μg L^−1^ for (*R*)-MDMA, respectively, and 0.096 and 1.01 μg L^−1^ for (*S*)-MDMA, respectively. Measured concentrations were within 10% of nominal concentrations. Thus, exposure levels were based on nominal concentrations.

#### 2.2.1. Morphophysiological Parameters

**Figure 4:**
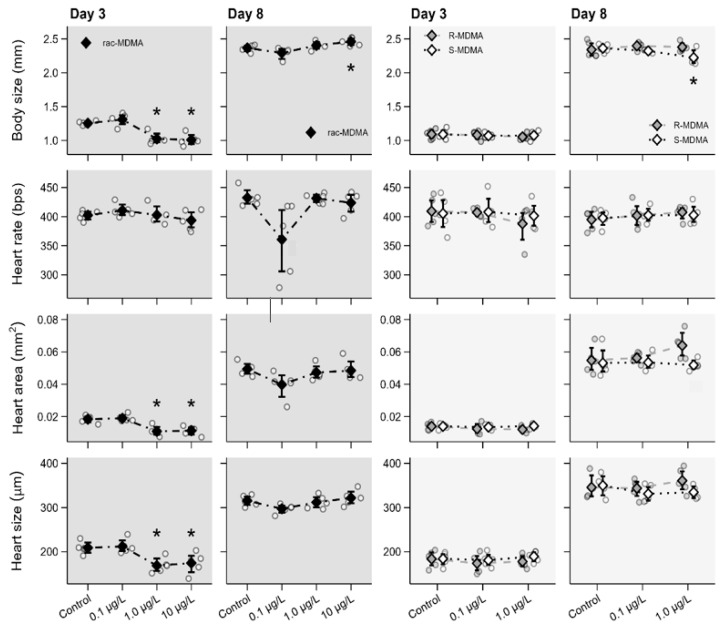
Morphophysiological effects of (*R*,*S*)-MDMA (**left**; dark grey panels) and its enantiomers (**right**; light grey panels) determined on days 3 and 8 in *Daphnia magna*. Data are presented as individual observations (circles), mean values (diamonds) and 95% bootstrap confidence intervals (error bars). Asterisks (*) represent significant differences relative to the control.

The racemate and both enantiomers interfered with the morphophysiology of juveniles and adults, and enantioselective effects were observed for body size. In detail, exposure to (*R*,*S*)-MDMA caused a significant decrease in body size, heart area and size at day 3 in juveniles exposed to the concentrations 1 µg L^−1^, i.e., 0.5 µg L^−1^ of each enantiomer, and 10 µg L^−1^, i.e., 5.0 µg L^−1^ of each enantiomer (Figure 4, Table S3). On the contrary, a significant increase in body size was found in adults at 10 µg L^−1^ (day 8), but no effects were observed in heart area and size at that age. No effects on heart rate were found in either juvenile or adult.

No changes in morphophysiological parameters were observed in the organisms exposed to either (*R*)- or (*S*)-enantiomer at day 3 (Figure 4, Table S3). However, an enantio-specific response was observed in the body size of organisms at day 8, demonstrated by a significant decrease of body size with exposure of 1 µg L^−1^ of (*S*)-MDMA in contrast to no change in body size for (*R*)-MDMA. No changes in heart rate, heart area and heart size at day 8 were observed for either juveniles and adults for both enantiomers.

Morphophysiological changes are consistent with our previous study with amphetamine (Ribeiro et al., submitted). In that study, adult daphnids exposed to (*S*)-amphetamine (AMP) also showed a significant decrease in body size whereas no changes were observed in the organisms exposed to (*R*)-AMP. Similarly, AMP racemate caused an increase in body size in adults although no effects were found in juveniles. (*R*,*S*)-MDMA also interfered with heart area and size but only in juveniles. No effects were found in adults. No enantioselective effects or changes were found in heart area and size or in heart rate for enantiomers for either juveniles or adults. No effects on morphophysiological parameters were found for (*R*)-MDMA. Nevertheless, careful analysis should be taken as both racemate and (*S*)-enantiomer interfered with morphophysiological parameters, even at environmental concentrations and at different development stages of the daphnia life cycle. Enantio-specificity was observed with the decrease of body size at exposure of 1 µg L^−1^ of (*S*)-MDMA.

#### 2.2.2. Swimming Behaviour

**Figure 5:**
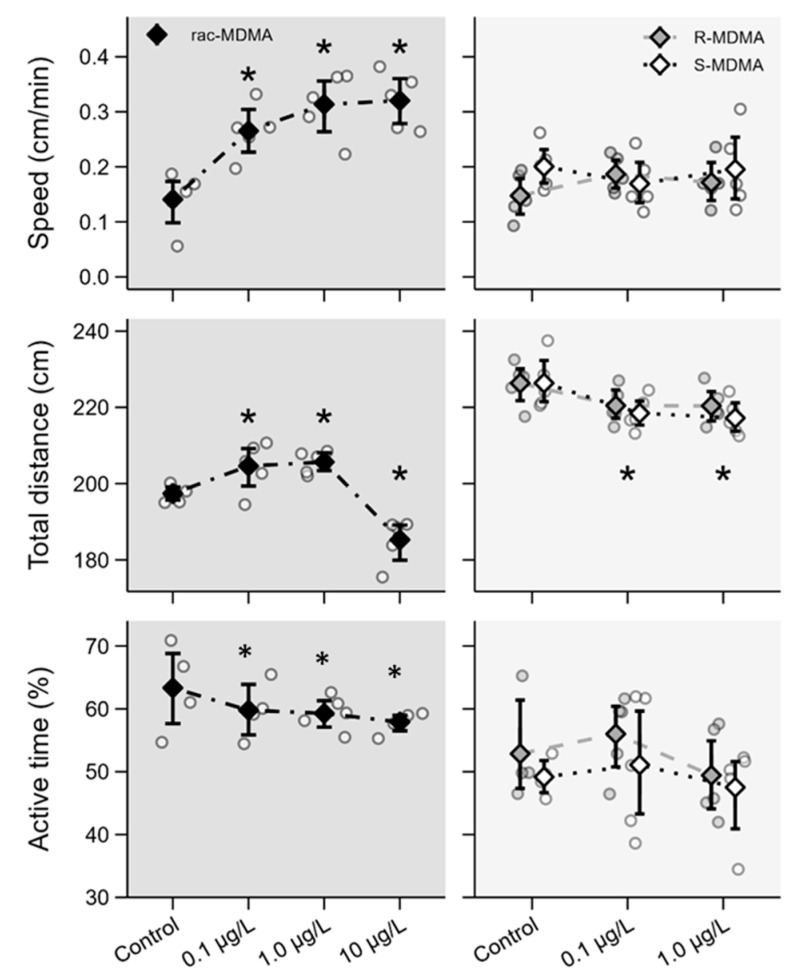
Behavioural effects of (*R*,*S*)-MDMA (**left**; dark grey panels) and its enantiomers (**right**; light grey panels) determined on day 5 in *Daphnia magna*. Data are presented as individual observations (circles), mean values (diamonds) and 95% bootstrap confidence intervals (error bars). Asterisks (*) represent significant differences relative to the control.

Behavioural responses are also important indicators of toxicity. Different responses were observed between the racemate and enantiomers, but enantioselective responses were not found. A significant increase in swimming speed was observed in the organisms exposed to the (*R*,*S*)-MDMA whereas individual enantiomers did not affect swimming speed (Figure 5, Table S4). Regarding the total distance travelled, different responses were observed for the racemate. Indeed, a significant increase was observed at 0.1 and 1 μg L^−1^, whereas a significant decrease was observed at 10 µg L^−1^ (Figure 5, Table S4). Both enantiomers elicited a significant decrease in total distance travelled at both concentrations, which were consistent across both enantiomers. Regarding active time, a significant decrease was observed for the racemate while no effects were observed for the enantiomers (Figure 5, Table S4).

Despite the increase in swimming speed and in the total distance travelled with exposure at environmental levels (0.1 and 1 μg L^−1^) of (*R*,*S*)-MDMA, a significant decrease in active time was noted and the enantiomers did not affect swimming speed, which can be associated with possible synergetic or competition effects of enantiomers that can enhance the effect of racemate in comparison to isolated enantiomers. Also, a significant decrease of total distance travelled was consistent across racemate (at the highest concentration) and both enantiomers. These results are in agreement with those obtained in the study developed by De Felice et al. [22] for cocaine, which showed that exposure to higher concentrations of the substance leads to a decrease in the distance travelled by daphnia, while exposure to lower concentrations leads to an increase in the distance travelled. A decrease in the total distance and an increase in avoidance were also reported in zebrafish larvae exposed to (*R*)-venlafaxine [12]. These results are corroborated by Stewart et al. [23], who demonstrated the inactivity of zebrafish exposed to high levels of MDMA (up to 10 mg L^−1^). Changes in swimming behaviour have been linked to changes in AChE activity. A decrease in AChE was found for (*R*,*S*)-MDMA only at the highest concentration (10 μg L^−1^), but no changes were noted at the lower concentrations and no changes in AChE were observed for either enantiomer.

#### 2.2.3. Reproductive Parameters

**Figure 6:**
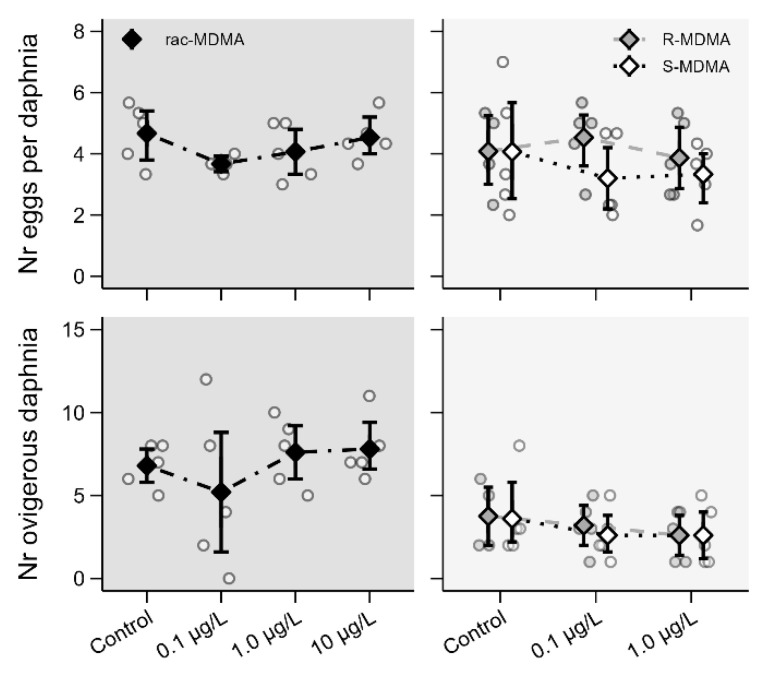
Reproductive effects of (*R*,*S*)-MDMA (**left**; dark grey panels) and its enantiomers (**right**; light grey panels) determined on day 8 in *Daphnia magna*. Data are presented as individual observations (circles), mean values (diamonds) and 95% bootstrap confidence intervals (error bars).

Reproductive parameters were not affected during sub-chronic exposure to the racemate and its enantiomers as no significant differences were observed in the number of ovigerous daphnia and the number of eggs per daphnia for either racemate or enantiomers (Figure 6, Table S5). However, it is important to stress that only first reproductive events were considered, which may conceal possible negative effects. Indeed, various studies have shown altered reproduction in daphnids exposed to PASs including AMP-like substances such as methamphetamine [24]. The 21-day reproduction assay should be performed for an accurate evaluation of the potential interference in reproductive events.

#### 2.2.4. Biochemical Parameters

**Figure 7:**
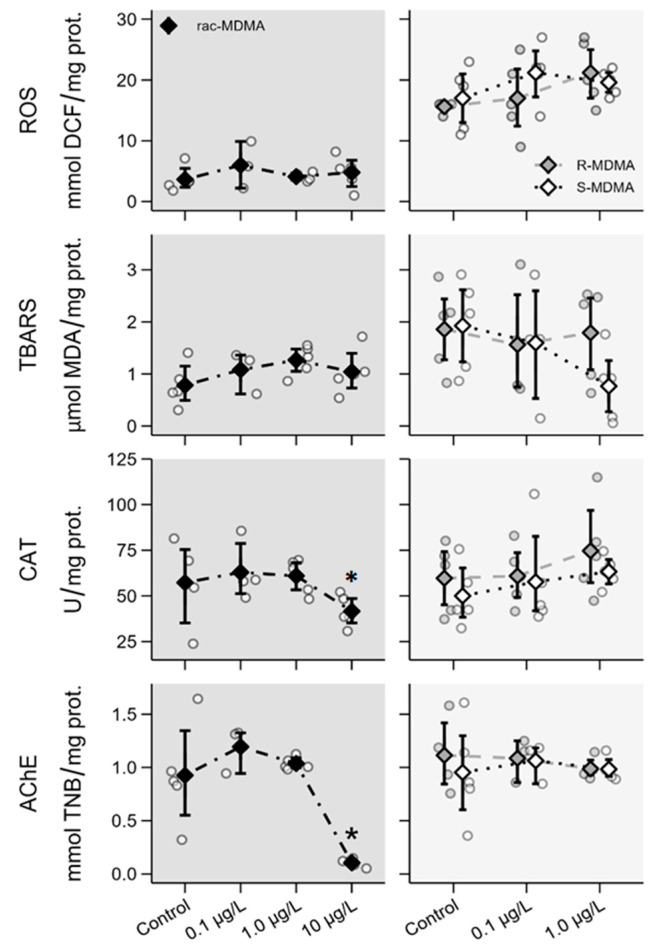
Biochemical effects of (*R*,*S*)-MDMA (**left**; dark grey panels) and its enantiomers (**right**; light grey panels) determined on day 8 in *Daphnia magna*. Data are presented as individual observations (circles), mean values (diamonds) and 95% bootstrap confidence intervals (error bars). Asterisks (*) represent significant differences relative to the control.

In biochemical parameters, no significant differences were found in (*R*,*S*)-MDMA and its enantiomers in most cases, except for a significant decrease in AChE and CAT activity in the highest concentration of the (*R*,*S*)-MDMA (10 μg L^−1^) (Figure 7, Table S6).

Some studies have shown that exposure to PASs induces oxidative stress and can affect the activity of several enzymes in non-target organisms even at low concentrations. Exposure of *D*. *magna* to benzoylecgonine at concentrations like those found in aquatic ecosystems induces oxidative stress and leads to the inhibition of AChE activity [25]; similarly, exposure to citalopram and mirtazapine increases levels of ROS and oxidative stress. In contrast, in our study, no significant changes were found in enzymatic levels, ROS and TBARS for the enantiomers. However, a significant decrease in AChE and CAT activity in daphnids exposed to the highest concentration of (*R*,*S*)-MDMA was observed. According to Parolini et al. [25], AChE activity is strictly related to behavioural changes in aquatic organisms. A reduction in AChE activity in aquatic organisms exposed to environmental pollutants has been attributed to oxidative stress [24]. Although a reduction in AChE enzymatic activity was observed at the highest concentrations (10 μg L^−1^) of the racemate, no increase in ROS levels was found; however, a decrease in CAT activity and altered swimming behaviour were observed within the range of concentrations tested and not only at the highest concentration of the racemate. Therefore, other mechanisms than AChE activity may be involved in changes observed in swimming behaviour. A similar experimental approach regarding endpoints and range of concentrations was performed with AMP (Ribeiro et al., submitted). Compared to AMP, MDMA seems to be less noxious to *D. magna* overall. Briefly, (*R*)-AMP affected body size and heart size in juveniles whereas no interferences in morphophysiological parameters were noted for (*R*)-MDMA in either juveniles or adults. Further, (*R*,*S*)-AMP as well as its enantiomers affected reproductive and biochemical parameters in contrast to the MDMA enantiomers. Only (*R*,*S*)-MDMA at the highest concentration interfered with enzymatic activity. Therefore, considering the selected parameters, MDMA seems to be less toxic to this organism in comparison to AMP. Nevertheless, (*R*,*S*)-MDMA and the (*S*)-enantiomer affected morphophysiological parameters, and the racemate and both its enantiomers affected swimming behaviour at reported environmental levels.

## 3. Materials and Methods

### 3.1. Chemicals and Materials

Hydrochloride MDMA racemate was acquired from Lipomed (Arlesheim, Switzerland). MDMA standard solution for enantioseparation was prepared in ethanol (EtOH) at a concentration of 30 mg mL^−1^ and stored at 4 °C. For ecotoxicity assays, stock solutions of the racemate and pure enantiomers (obtained by semipreparative chromatography) were prepared at 1 mg mL^−1^ in EtOH and then at 1 mg L^−1^ with ultra-pure water (UPW).

UPW was obtained from an Ultrapure Water System (SG Ultra Clear UV plus). Solvents used for chromatography were of chromatographic grade. EtOH (≥99.8%), methanol (MeOH) and isopropanol (IPA) were acquired from Fisher Scientific UK (Leicestershire, United Kingdom); *n*-hexane (*n*-Hex, ≥97.0%) was acquired from VWR BDH Chemicals (Gliwice, Poland); diethylamine (DEA, 99.5%) and diethyl ether were acquired from Sigma-Aldrich (Co, Belgium); hydrogen chloride solution in diethyl ether were acquired from Alfa Aesar (Thermofisher, Kandel, Germany); ammonium acetate was purchased from Sigma-Aldrich (Zwijndrecht, The Netherlands); ammonium bicarbonate was acquired from Sigma-Aldrich (Darmstadt, Germany); pure anhydrous sodium sulphate 99.7% was acquired from José Manuel Gomes dos Santos, LDA (Odivelas, Portugal).

For the chemical analysis of MDMA in culture media, the chiral reagent (*R*)-(−)-α-methoxy-α-(trifluoromethyl) phenylacetyl chloride [(*R*)-MTPA-Cl], sodium hydroxide (NaOH), ammonium hydroxide (NH_4_OH) 25%, triethylamine (TEA) and sulfuric acid (H_2_SO_4_) were purchased from Sigma-Aldrich (Steinheim, Germany). Oasis MCX 150 mg (6 cc) solid-phase extraction (SPE) cartridges were purchased from Waters (Dublin, Ireland). Glass microfibers filter with 0.7 μm porous size was purchased from VWR (Leuven, Belgium).

All chemicals used for culture media and biochemical assays were of higher analytical purity and described in the following sections.

### 3.2. Equipment and Chromatographic Conditions

A high-performance liquid chromatography with a diode array detector equipment (HPLC-DAD) from LaChrom Merck Hitachi^®^, equipped with an interface system (D-7000), a DAD (L-7455), a pump (L-7100), an autosampler (L-7200) and a data acquisition software (System Manager HSMP-7000, Version 3.0), was used for semipreparative enantioresolution of (*R*,*S*)-MDMA. Chromatographic separation was performed according to the method previously developed by Gonçalves et al. [20]. Briefly, the chiral stationary phase used was a homemade amylose 3,5-dimethylphenylcarbamate column coated with APS-Nucleosil (500 Å, 7 µm, 20%, *w*/*w*; 20 cm × 0.7 cm internal diameter). The enantioseparation was performed under normal elution mode with *n*-Hex with 0.1% DEA and EtOH (80:20, *v*/*v*) as mobile phase, at room temperature, with a flow rate of 1.5 mL min^−1^ and the DAD detector adjusted to a wavelength of 210 nm. Collected fractions were evaporated to dryness in a water bath at approximately 35–37 °C, solubilized in IPA followed by precipitation with HCl in ether dropwise and diethyl ether. The procedure was repeated several times to achieve the maximum recovery of the enantiomers. The precipitate of the hydrochloride enantiomers was collected and reconstituted in EtOH. Enantiomeric purity of the fractions was evaluated using the same equipment and chromatographic conditions. The elution order of each enantiomer, under these conditions, was determined in our previous work [20].

For quantification of enantiomers in each fraction collected in semipreparative enantioresolution, an analytical chromatography methodology was developed using a Shimadzu UFLC Prominence system equipped with a column oven (CTO-20AC), a system controller (CBM-20A), 2 pumps (LC-20AD), an autosampler (SIL-20AC), a data acquisition software LC Solution, version 1.24 SP1 (Shimadzu Corporation, Tokyo, Japan), and a Shimadzu SPD-20A UV/Vis detector coupled to the LC system; Lux^®^ 3 µm i-Amylose-3 column (LC Column 150 × 2.0 mm) (Phenomenex, Torrance, CA, USA); EtOH and Ultrapure water (UPW) with 0.1% DEA as mobile phase; detector at 210 nm; flow rate of 0.1 mL min^−1^; and sample injection volume of 10 μL.

### 3.3. Chemical Analysis of MDMA in Culture Media

The MDMA racemate and enantiomers concentration levels in culture media were confirmed by an adapted GC-MS method [2]. For that, the pre-filtered and acidified culture medium (250 mL) was pre-concentrated by solid-phase extraction (SPE) with OASIS MCX (150 mg, 6 cc) cartridges [2]. The SPE extracts were dried, reconstituted in 200 μL water and derivatized using the chiral reagent (*R*)-MTPA-Cl according to our previous procedure [2]. A matrix-matched calibration curve in culture media was constructed from 80 to 320 ng L^−1^. Linear parameters and limit of quantification (LOQ) can be found as Supplementary Materials (SM) (Table S1). For measurement of (*R*,*S*)-MDMA at 1 μg L^−1^ and 10 μg L^−1^, as well as enantiomers at 1 μg L^−1^, a dilution factor was applied.

### 3.4. Daphnia Magna Cultures

Monoclonal cultures of *D. magna* were maintained at 20 ± 2 °C and a 16:8 h light/dark cycle in moderately hard reconstituted water (MHRW) prepared using the following chemicals: 123 mg L^−1^ magnesium sulphate heptahydrate (MgSO_4_·7H_2_O, >99%) and 60 mg L^−1^ calcium sulphate dihydrate (CaSO_4_·2H_2_O, >99%) obtained from Merck (Darmstadt, Germany); 96 mg L^−1^ sodium bicarbonate (NaHCO_3_, ≥99%) purchased from Sigma-Aldrich (St. Louis, MO, USA); and 4 mg L^−1^ potassium chloride (KCl, >99%) obtained from Panreac (Barcelona, Spain).

Before being used, MHRW medium was aerated for about 30 min with an air diffuser (coupled to an air pump) under continuous magnetic agitation; pH and conductivity were measured using the multiparameter analyser HANNA Consort C863 (Turnhout, Belgium). The medium was supplemented with 9 mL of an *Ascophyllum nodosum* extract stock solution from SOL-PLEX^®^ SIERRA|Alltech (Lexington, KY, USA) and a vitamin mixture solution (50 μL): cyanocobalamin (B12, >98.9%) purchased from Fragon Iberian Laboratory (Oporto, Portugal), biotin (H, ≥99%) purchased from Panreac AppliChem ITW Reagents (Darmstadt, Germany) and thiamine HCl (B1) purchased from Couto pharmacy manipulation laboratory (Oporto, Portugal) per L. Organisms were fed with a suspension of the microalgae *Raphidocelis subcapitata* at 3.0 × 10^5^ cells mL^−1^ or 6.0 × 10^5^ cells mL^−1^ for juveniles and adults (egg-bearing females), respectively, supplemented with 500 μL of an extract of dried yeast (Pura Vida, Lisbon, Portugal). Microalgae were cultured in Woods Hole MBL medium in a semicontinuous 4 L batch culture at 20 C ± 2 °C in a 16:8 h light/dark cycle. Details about the culture maintenance of microalgae are described in the SM.

### 3.5. Ecotoxicity Assay: Experimental Design and Procedures

Each experimental unit (vessel) consisted of a glass flask with 200 mL of MHRW medium with 15 daphnia born between the 3rd and 5th clutches. Experiments were initiated with <24-hours-old organisms (neonates) using 5 replicate vessels per concentration and negative control (0 µg L^−1^). Organisms were exposed to environmental and sublethal concentrations of MDMA racemate at three concentration levels (0.1, 1 and 10 µg L^−1^) and each enantiomer at two concentration levels (0.1 and 1 µg L^−1^) for 8 days at 20 °C ± 2 °C and in a 16:8 h light/dark cycle. At 48 h intervals, test solutions were renewed and organisms were fed with microalgae at a ratio of 3.0 × 10^5^ cells mL^−1^ for neonates and juveniles (from the start of the assay until day 4) or 6.0 × 10^5^ cells mL^−1^ for adults (from day 4 to day 8).

The ecotoxicity assay was designed to accommodate various endpoints, including morphophysiology (body size, heart area, heart size and heart rate), swimming behaviour (swimming speed, distance travelled and active time), reproduction (number of ovigerous daphnia and number of eggs per daphnia) and biochemical parameters (oxidative stress and enzymatic biomarkers).

Three daphnids per replicate were randomly collected for morphophysiological endpoints assessment on days 3 and 8 of the experiment. Each daphnid was placed on a microscope slide with a drop of the corresponding test solution, photographed and video recorded for 75 s using a digital camera (Canon PowerShot G9) attached to the microscope. Daphnids were returned to the corresponding vessel and body size and heart parameters (rate, area and size) were determined using free image analysis software.

Swimming behaviour was determined on day 5 using video recordings of six random daphnia per replicate vessel. For that, a 6-well plate was previously filled with melted 1% agarose. After solidification, the agarose was cut using a plastic ring with a diameter of about 27 mm to create a circular swimming arena, filled with 5 mL of test medium. Organisms were acclimated for ten minutes in the arena and then the plates were set up on top of a white screen for video capture with a perpendicularly mounted digital camera (Canon Legria HF R506) with a resolution of 30 frames per second. Daphnids were then transferred back to the appropriate vessel. The Real Trackfish software was used to analyse the videos and determine swimming speed, distance travelled and active time [12,26].

Reproductive status was checked at day 8. For that, three random daphnia per replicate vessel were photographed and the number of eggs in the brood pouch was determined. Additionally, the number of ovigerous daphnia (egg-carrying females) per vessel was determined. At the end of the exposure, all daphnia from each replicate vessel were collected into an Eppendorf tube, washed with cold buffer (0.800 g NaCl, 0.020 g KCl, 0.144 g Na_2_HPO_4_ and 0.024 g KH_2_PO_4_ in 100 mL of UPH_2_O) and refrigerated at −80 °C until biochemical analysis (details of chemicals and reagents can be found in SM).

### 3.6. Ecotoxicity Assay: Biomarker Quantification

Daphnia tissues were ultrasonically homogenized in 250 μL of buffer and centrifuged at 13,000× *g* for 20 min at 4 °C. The supernatant was collected for analysis of catalase (CAT) and acetylcholinesterase (AChE) activities, reactive oxygen species (ROS) and lipid peroxidation (TBARS—thiobarbituric acid-reactive substances) [24,27,28]. Each sample was analysed in duplicate.

Coomassie Plus—The Better Bradford Assay Reagent was used to determine the presence of proteins according to the Coomassie blue technique, using a bovine serum albumin calibration curve and a microplate reader to measure absorbance at 595 nm [29].

AChE activity was determined based on Ellman’s method, which monitors 5-thio-2-nitrobenzoic acid (TNB) production at 412 nm [30,31] using a microplate reader (3 min kinetics at 25 °C) and considering a coefficient of extinction of 14.3 mL mmol^−1^ cm^−1^; results are expressed as mmol TNB/mg protein.

Enzymatic activity of CAT was determined at 414 nm and 25 °C based on the conversion of H_2_O_2_ into H_2_O and O_2_ [32,33]. CAT activity was expressed as UCAT/mg protein.

Using a fluorescent probe (2,7-dichlorofluorescin diacetate) and a calibration curve with 2′,7′-dichlorofluorescein (DFC), ROS levels were measured based on the method of Deng et al. [34] using a 96-well microplate reader and excitation and emission wavelengths of 485 nm and 528 nm, respectively. The results were given in μmol of DFC/mg protein.

For TBARS, malondialdehyde (MDA) levels were determined using the thiobarbituric acid colorimetric method [35]. The amounts of MDA were measured using an MDA standard curve. Samples were heated to 60 °C for 40 min and then cooled for 15 min on ice. After that, 10 μL of 20% sodium dodecyl sulphate (SDS) were added and the absorbance read at 530 nm. Results were presented as μmol MDA/mg protein.

### 3.7. Statistical Analysis

Data from the racemate (*R*,*S*)-MDMA and enantiomers were analysed as separate (i.e., independent) experiments.

To assess significant effects of (*R*,*S*)-MDMA concentrations on the endpoints evaluated in *D. magna*, generalised linear models (glm) were used with concentration as a categorical predictor (unifactorial design). For morphophysiological, behavioural and biochemical endpoints, data were modelled as normally distributed (linear model), whereas reproductive endpoints were modelled as count data (negative binomial glm)—for further insight, see [36]. In both cases, the presence of a significant effect of concentration was further examined with Dunnett contrasts to assess significant differences between treatment and control (0 µg/L). Residual plots were used to critically analyse model fit (removal of outliers or verification of normality and homoscedasticity, when applicable).

An analogous approach was used for (*R*)-MDMA and (*S*)-MDMA. In this case, however, both enantiomer and concentration were used as categorical predictors (bifactorial design) in the generalised linear models, which allowed to test the interaction between concentration and the enantiomeric form (i.e., statistical evidence of the presence of enantioselectivity). As above, morphophysiological, behavioural and biochemical endpoints were modelled as normally distributed data (linear model) and reproductive endpoints were modelled as count data (negative binomial glm). Again, residual plots were used to critically analyse model fit. In the case of a significant enantiomer × concentration interaction, simple main effects [37] of concentration were tested separately for each enantiomer. Significant differences relative to the control were analysed with Dunnett contrasts (separately for each enantiomer or jointly, depending on the presence or not of an enantiomer × concentration interaction).

Statistical analyses were performed with jamovi v. 2.2.5 [38] using a significance level of 0.05. Plots were built with R v. 4.2.1 [39] using the graphical interface RStudio v. 2022.7.1.554 [40]; R packages ggplot2 [41], cowplot [42] and envalysis [43] were required.

## 4. Conclusions

The possible approval of MDMA to support PTSD treatment might increase its levels in WWTPs and surface waters. Thus, the investigation on its potential enantioselective toxicity is necessary for an adequate risk assessment of these substances.

Enantiomers were isolated by a chiral semipreparative method previously established by our team for further ecotoxicity assays. Both enantiomers of MDMA were achieved at high enantiomeric purity.

Different sensitive endpoints of toxicity were selected to provide a comprehensive evaluation of MDMA enantiotoxicity and assess ecological risks. For some parameters, different responses were observed between the racemate and enantiomers. This can be due to possible synergetic or competition effects between enantiomers that can cause a different response of the mixture in comparison with single enantiomers. The results showed that exposure to MDMA racemate and enantiomers, at reported environmental concentrations, can interfere with morphophysiological characteristics and swimming behaviour responses, and modulate AChE activity (10 μg L^−1^) in *D. magna*. Body size and heart area showed enantioselective effects over time. Our results suggest that the (*R*)-enantiomer is less toxic than the (*S*)-enantiomer. Though the (*R*)-enantiomer is excreted in higher amounts, MDMA has been found at different enantiomeric fractions as illicit discharges during apprehension episodes might lead to the occurrence of the racemate.

No effects on the first reproductive events and biochemical parameters (other than AChE and CAT) were found, suggesting that MDMA does not interfere with these parameters.

Since MDMA is expected to increase in the aquatic environment, with potential consequences for aquatic organisms and water quality, environment directives must take this into account and adopt measures to mitigate the impact of these substances on the environment and reduce the impacts on wildlife and humans.

## Data Availability

Data are available from the corresponding author upon request.

## Supplementary Materials

The following supporting information can be downloaded at: https://www.mdpi.com/article/10.3390/molecules28031457/s1, Table S1: Linearity parameters, method LOQ and recovery; Table S2: Linear parameters of MDMA enantiomers calibration curve and recovery; Table S3. Effects of (*R*,*S*)-MDMA and its enantiomers on the morphophysiological parameters determined in *Daphnia magna* on days 3 and 8. Significant effects are highlighted in bold (*p* ≤ 0.05). In the experiment with (*R*)- and (*S*)-MDMA, a significant interaction between enantiomer and concentration reveals an enantioselective response; Table S4. Effects of (*R*,*S*)-MDMA and its enantiomers on swimming behaviour parameters determined in *Daphnia magna* on day 5. Significant effects are highlighted in bold (*p* ≤ 0.05). In the experiment with (*R*)- and (*S*)-MDMA, a significant interaction between enantiomer and concentration reveals an enantioselective response; Table S5. Effects of (*R*,*S*)-MDMA and its enantiomers on reproductive parameters determined in *Daphnia magna* on day 8. Significant effects are highlighted in bold (*p* ≤ 0.05). In the experiment with (*R*)- and (*S*)-MDMA, a significant interaction between enantiomer and concentration reveals an enantioselective response; Table S6. Effects of (*R*,*S*)-MDMA and its enantiomers on biochemical parameters determined in *Daphnia magna* on day 8. Significant effects are highlighted in bold (*p* ≤ 0.05). In the experiment with (*R*)- and (*S*)-MDMA, a significant interaction between enantiomer and concentration reveals an enantioselective response.
