# Supplementary material for: Toxicity of the 3,4-Methylenedioxymethamphetamine and Its Enantiomers to Daphnia magna after Isolation by Semipreparative Chromatography
Source: Molecules. 2023 Feb 2;28(3):1457. doi: 10.3390/molecules28031457 (PMC9920394; doi:10.3390/molecules28031457)
Supplement: Supplementary file 1 [file molecules-28-01457-s001.zip › molecules-2161981-supplementary.pdf]

Supplementary material

Toxicity of the 3,4-methylenedioxymethamphetamine (MDMA) and its enantiomers to *Daphnia magna*, after their isolation by semipreparative chromatography

Ana Rita Costa[1], Virgínia M.F. Gonçalves[1,2], Bruno Castro[3,4], João Carrola[5,6], Maria E. Tiritan[1,7,8*], Ivan Langa[1], Ariana Pereira[1,6], Ana Rita Carvalho[1], Cláudia Ribeiro[1,7*]

1. Experimental

1.1. Culture maintenance of microalgae

An algae culture medium composed by macronutrients and micronutrients was prepared with the following chemicals: $MgSO_4.7H_2O$, and boric acid (H_3BO_3 , ≥99.8%) both obtained from Merk (Darmstadt, Germany); Manganese(II) chloride hexahydrate ($MnCl_2.4H_2O$, ≥98%) and cobalt(II) chloride hexahydrate ($CoCl_2.6H_2O$, >99%) purchased from PA Panreac (Barcelona, Spain); Zinc chloride ($ZnCl_2$, >97%) and Potassium hydrogen phosphate (K_2HPO_4, >99%) acquired from Panreac AppliChem ITW Reagents (Darmstadt, Germany); Sodium nitrate ($NaNO_3$,>99%), Sodium bicarbonate ($NaHCO_3$, ≥ 99,7 %), Sodium molybdate dihydrate ($Na_2MoO_4.2H_2O$, ≥98%) and Disodium EDTA dihydrate ($Na_2EDTA.2H_2O$, ≥98.5%) acquired from Sigma-Aldrich (Missouri, USA); Magnesium Chloride Hexahydrate ($MgCl_2.6H_2O$, >98%) and Calcium chloride dihydrate ($CaCl_2.2H_2O$, ≥ 99%) were acquired from Riedel-de-Haën (North Caroline, USA); and Iron(III) Chloride Hexahydrate ($FeCl_3.6H_2O$,≥ 97%) was purchased from PRS Panreac (Barcelona, Spain). After preparation, the culture medium is aerated for 60 minutes and inoculated with *R. subcapitata* and maintained for 8 days, at constant uniform light (6000 lux for bottom illumination), 20 ± 2ºC and an air pump system with continuous magnetic agitation. After 8 days of incubation, 250 mL of the microalgae culture was used for the next culture. The remainder of the microalgae culture was centrifuged at 3000 rpm for 10 minutes, the supernatant discarded, and the algae pellet reconstituted in MHRW medium. The optical density (OD) of algae suspension was measured at a wavelength of 440 nm and adjusted to 0.6-0.8. The algae suspension was kept at 4ºC and used as food for the daphnia. An autoclave from PBI (South Carolina, USA) and a laminar flow chamber SC4 from Allentown (New Jersey, USA) were used

for the preparation and manipulation of solutions and media. Absorbance was measured using an UV/Vis spectrometer (ATI Unicam, Leeds, England).

1.2. Chemicals used for biochemical determinations

For biochemical determinations the following reagents were acquired from Sigma-Aldrich (Missouri, USA): Sodium chloride (NaCl); potassium chloride (KCl); disodium phosphate (Na_2HPO_4); potassium dihydrogen phosphate (KH_2PO_4); Coomassie Plus (The Better Bradford AssayTM Reagent); bovine serum albumin (BSA, ≥96%); UPW; tris base; hydrochloric acid 37%; 5,5'-dithiobis-2-nitrobenzoic acid (DTNB, ≥ 98%); acetylthiocholine iodide (ATCI, ≥ 99%); 2,7-dichlorofluorescein (DCF, 90%); 2,7-dichlorofluorescein diacetate (H_2DCFDA, ≥ 97%); dimethyl sulfoxide (DMSO, ≥ 99.9%); monosodium phosphate (NaH_2PO_4, ≥ 99%; ammonium molybdate tetrahydrate (AMT); butylated hydroxytoluene (BHT, ≥ 99%); thiobarbituric acid (TBA, ≥98%); sodium dodecyl sulphate (SDS, ≥ 98.5%); trichloroacetic acid (TCA); malondialdehyde (MDA, ≥ 96%) and EtOH 99.8%. For adjustment of pH, the solutions 6M of sodium hydroxide (NaOH) and 0.5M chloridric acid (HCl) were used.

An Inverse Microscope of ZEISS (Jena, Germany) and a Neubauer chamber for microalgae cell counting. A microplate reader, BioTek Synergy 2 (Vermont, USA) was used for biochemical analysis and an ultrasonic of VWR USC-TH (Pennsylvania, USA) for preparation of the daphnia homogenates.

1.3. Chemical analysis of MDMA in culture medium

Table S1 - Linearity parameters, method LOQ and recovery.

PAS	Concentration range (ng L^{-1})	Equation	r^2	LOQ (ng L^{-1})	Recovery (%)
(*R*) -MDMA	80.0 - 320	y=98.623x + 1332.3	0.9984	80.0	90.0 – 111
(*S*)-MDMA	80.0 - 320	y=96.534x + 1296.2	0.9988	80.0	93.8 – 109

2. Results and Discussion

2.1. Analytical method parameters for determination of the recovery of the enantiomers obtained by semipreparative chromatography

Table S2- Linear parameters of MDMA enantiomers calibration curve and recovery.

Enantiomer	Range (µg mL^{-1})	Linear Equation	R^2	Concentration of the enantiomers (µg mL^{-1})	Semi-preparative enantiomers recovery (%)
(*R*)-MDMA	5.0 – 50.0	Y=229471x+158183	0.9996	6095.8	40.7
(*S*)-MDMA	5.0 – 75.0	Y=224541.08x+277228.35	1.0000	293.2	2.0

2.2. Ecotoxicity assay

Table S3. Effects of (*R*,*S*)-MDMA and its enantiomers on the morphophysiological parameters determined in *Daphnia magna* on days 3 and 8. Significant effects are highlighted in bold ($p \leq 0.05$). In the experiment with (*R*)- and (*S*)-MDMA, a significant interaction between enantiomer and concentration reveals an enantioselective response.

Variable	Source of variation	Day 3			Day 8		
		d.f.	*F*	*p*	d.f.	*F*	*p*
**Body size (µm)**	(*R*,*S*)	3, 16	20.4	**<0.001**	3, 15	5.16	**0.012**
	Enantiomer	1, 24	0.0212	0.885	1, 22	11.2	-
	Concentration	2, 24	0.506	0.609	2, 22	3.95	-
	Interaction	2, 24	0.204	0.817	2, 22	6.21	**0.007**
**Heart rate (bpm)**	(*R*,*S*)	3, 16	1.29	0.313	3, 14	2.02	0.158
	Enantiomer	1, 24	0.146	0.706	1, 24	0.00813	0.929
	Concentration	2, 24	0.899	0.420	2, 24	0.781	0.469
	Interaction	2, 24	0.335	0.718	2, 24	0.154	0.858
**Heart area (µm^2)**	(*R*,*S*)	3, 16	14.3	**<0.001**	3, 16	2.66	0.083
	Enantiomer	1, 24	2.03	0.167	1, 23	3.88	0.061
	Concentration	2, 24	0.661	0.525	2, 23	0.384	0.688
	Interaction	2, 24	0.707	0.503	2, 23	0.992	0.386
**Heart size**	(*R*,*S*)	3, 16	7.87	**0.002**	3, 16	2.76	0.076

**(µm)**	Enantiomer	1, 24	1.129	0.298	1, 21	1.587	0.222
	Concentration	2, 24	0.508	0.608	2, 21	0.607	0.554
	Interaction	2, 24	0.303	0.741	2, 21	0.867	0.435

d.f. = degrees of freedom; F =value of statistical test; *p*-probability (statistical differences ≤ 0.05)

Table S4. Effects of (*R*,*S*)-MDMA and its enantiomers on swimming behaviour parameters determined in *Daphnia magna* on day 5. Significant effects are highlighted in bold ($p \leq 0.05$). In the experiment with (*R*)- and (*S*)-MDMA, a significant interaction between enantiomer and concentration reveals an enantioselective response.

**Variable**	**Source of variation**	**d.f.**	***F***	***p***
**Speed (cm/min)**	(*R*,*S*)	3, 16	12.5	**<0.001**
	Enantiomer	1, 24	1.25	0.274
	Concentration	2, 24	0.0949	0.910
	Interaction	2, 24	1.33	0.282
**Total distance travelled (cm)**	(*R*,*S*)	3, 16	19.5	**<0.001**
	Enantiomer	1, 24	0.804	0.379
	Concentration	2, 24	6.31	**0.006**
	Interaction	2, 24	0.236	0.791
**Active time (%)**	(*R*,*S*)	3, 13	4.05	**0.031**
	Enantiomer	1, 22	1.46	0.240
	Concentration	2, 22	1.10	0.349
	Interaction	2, 22	0.0977	0.907

d.f. = degrees of freedom; F = value of statistical test; *p*-probability (statistical differences ≤ 0.05)

Table S5. Effects of (*R*,*S*)-MDMA and its enantiomers on reproductive parameters determined in *Daphnia magna* on day 8. Significant effects are highlighted in bold ($p \leq 0.05$). In the experiment with (*R*)- and (*S*)-MDMA, a significant interaction between enantiomer and concentration reveals an enantioselective response.

**Variable**	**Source of variable**	**d.f.**	χ^2	***p***
**Nº daphnia with eggs**	(*R*,*S*)	3, 16	3.20	0.362
	Enantiomer	1, 23	0.149	0.700
	Concentration	2, 23	1.85	0.396
	Interaction	2, 23	0.171	0.918

	(*R*,*S*)	3, 15	0.666	0.881
**Nº eggs per daphnia**	Enantiomer	1, 23	0.764	0.382
	Concentration	2, 23	0.289	0.866
	Interaction	2, 23	0.556	0.757

d.f. = degrees of freedom; χ^2= value of statistical test; *p*-probability (statistical differences ≤ 0.05)

Table S6. Effects of (*R*,*S*)-MDMA and its enantiomers on biochemical parameters determined in *Daphnia magna* on day 8. Significant effects are highlighted in bold (p ≤ 0.05). In the experiment with (*R*)- and (*S*)-MDMA, a significant interaction between enantiomer and concentration reveals an enantioselective response.

**Variable**	**Source of variation**	***d.f.***	***F***	***p***
**ROS (μmol DCF/mg protein)**	(*R*,*S*)	3, 13	0.638	0.604
	Enantiomer	1, 24	0.669	0.421
	Concentration	2, 24	2.20	0.132
	Interaction	2, 24	1.06	0.363
**CAT (U/mg protein)**	(*R*,*S*)	3, 13	4.39	**0.024**
	Enantiomer	1, 22	2.50	0.128
	Concentration	2, 22	1.41	0.260
	Interaction	2, 22	0.513	0.605
**TBARS (μmol MDA/mg protein)**	(*R*,*S*)	3, 14	1.36	0.296
	Enantiomer	1, 22	0.781	0.386
	Concentration	2, 22	1.12	0.344
	Interaction	2, 22	1.12	0.346
**AChE (mmol TNB/mg protein)**	(*R*,*S*)	3, 14	48.6	**<0.001**
	Enantiomer	1, 16	0.0639	0.804
	Concentration	2, 16	1.08	0.361
	Interaction	2, 16	0.0125	0.988

d.f. = degrees of freedom; F = value of statistical test; *p*-probability (statistical differences ≤ 0.05)
